# Subclass responses and their half-lives for antibodies against EBA175 and PfRh2 in naturally acquired immunity against *Plasmodium falciparum* malaria

**DOI:** 10.1186/1475-2875-13-425

**Published:** 2014-11-05

**Authors:** Hodan Ahmed Ismail, Muyideen K Tijani, Christine Langer, Linda Reiling, Michael T White, James G Beeson, Mats Wahlgren, Roseangela Nwuba, Kristina EM Persson

**Affiliations:** Department of Microbiology, Tumor and Cell Biology (MTC), Karolinska Institutet, Nobels väg 16, 17177 Stockholm, Sweden; Cellular Parasitology Programme, Cell Biology and Genetics Unit, Department of Zoology, University of Ibadan, Ibadan, Nigeria; The Macfarlane Burnet Institute for Medical Research and Public Health, Melbourne, VIC Australia; Department of Infectious Disease Epidemiology, Imperial College, London, UK; Department of Laboratory Medicine, Lund University, University Hospital, Lund, Sweden

**Keywords:** *Plasmodium falciparum*, Malaria, EBA175, PfRh2, Merozoite, IgG, Subclass, Antibody, HbAA, HbAS

## Abstract

**Background:**

*Plasmodium falciparum* EBA175 and PfRh2 belong to two main families involved in parasite invasion, and both are potential vaccine candidates. Current knowledge is limited regarding which target antigens and subclasses of antibodies are actually important for protection, and how naturally acquired immunity is achieved.

**Methods:**

Repeated blood samples were collected from individuals in Nigeria over a period of almost one year. ELISA was used to analyse subclasses of IgG responses.

**Results:**

For both EBA175 (region III-V) and (a fragment of) PfRh2, the dominant antibody responses consisted of IgG1 and IgG3 followed by IgG2, while for PfRh2 there was also a relatively prominent response for IgG4. High levels of IgG1, IgG2 and IgG3 for EBA175 and total IgG for PfRh2 correlated significantly with a lower parasitaemia during the study period. Children with HbAS had higher levels of some subclasses compared to children with HbAA, while in adults the pattern was the opposite. The half-lives of IgG2 and IgG4 against EBA175 were clearly shorter than those for IgG1 and IgG3.

**Conclusion:**

EBA175 and PfRh2 are potential targets for protective antibodies since both correlated with lower parasitaemia. The shorter half-lives for IgG2 and IgG4 might explain why these subclasses are often considered less important in protection against malaria. Triggering the right subclass responses could be of critical importance in a successful vaccine. Further studies are needed to evaluate the role of haemoglobin polymorphisms and their malaria protective effects in this process.

## Background

The protozoa *Plasmodium falciparum* still remains a major global health problem. It is a leading cause of death among children under the age of five and pregnant women in sub-Saharan Africa [1, 2]. The increasing problem of drug resistance and the limited effect of vector control interventions make a call for a malaria vaccine urgent [3].

The *P. falciparum* blood stage invasion involves many complex interactions between merozoite antigens and erythrocyte receptors. There are two main merozoite invasion families: erythrocyte binding-like (EBL) proteins and reticulocyte binding protein homologue (RBP/PfRh) proteins [4, 5]. The EBL proteins include the erythrocyte binding antigens (EBAs), which are found in the micronemes of the merozoite and include EBA140, EBA175 and EBA181. The PfRh proteins are located in the rhoptries of the merozoites and include PfRh1, PfRh2a/2b, PfRh4, and PfRh5 [4–8]. PfRh2a and PfRh2b share the same amino acids for the first 88% of the protein [9]. EBA175 and PfRh2 are representatives of the sialic acid-dependent and non-sialic acid-dependent invasion pathways, respectively [10, 11], and both are potential vaccine candidates. EBA175 binds to glycophorin A on the erythrocyte surface [4], but the receptor for PfRh2 is not yet known.

Acquired immunity to malaria develops only after repeated exposure in individuals living in endemic areas, and it has been suggested that young children are less able to induce long-lived antibody secreting cells [12–15]. However, it seems that some antibodies, such as those against MSP1, can have a half-life that spans over many years [16]. It is known that antibodies are an important component of acquired immunity, and it has been shown that passive transfer of antibodies from immune donors to individuals with *P. falciparum* infections reduced parasitaemia and clinical symptoms [17]. Antibodies against several merozoite antigens, including EBAs and PfRhs, have been shown to be associated with protection against malaria in prospective longitudinal studies [13, 18–21]. Cytophilic immunoglobulins, IgG1 and IgG3 have been considered more important for protection as non-cytophilic IgG2 and IgG4 may block the protective activity of the cytophilic antibodies [20–25]. IgG1 and IgG3 are believed to neutralize parasites directly by inhibiting the parasite, or indirectly by opsonization [24–27].

There are several genetic polymorphisms that have been described to be protective against the severe forms of malaria, and one of these is sickle haemoglobin (HbS), of which there is a high prevalence in sub-Saharan Africa [28–30]. In the homozygous form it can be deleterious to the individual, but protective against malaria in the heterozygous form (HbAS), as described over 60 years ago [31]. Since then, several studies have shown the protective effects of HbAS on malaria [30, 32, 33]. A recent meta-analyses study of children with HbAS showed more than 90% protection from severe malaria [33]. Other studies have shown HbAS to give 30-50% protection from uncomplicated malaria [30, 33–36]. The mechanism behind the protective effect is still unclear, but it probably involves both the impaired development of the parasite inside the erythrocyte and a better immunological response, and it has also been suggested that a mechanism of impaired cytoadherence could be of importance [37].

In this study, naturally acquired antibody responses, total IgG as well as IgG subclasses, were investigated against EBA175 and PfRh2, as representatives of two different invasion pathways. Combinations of the two antigens together have been suggested to be more effective as vaccines, compared to using individual antigens [38]. Furthermore, associations between different haemoglobin genotypes and the presence of acquired antibodies against these antigens were investigated. Samples used were collected at least once a month from children and adults living in Igbo-Ora, a rural area of Nigeria, over a period of almost one year. Very few studies have looked at immunoglobulin subclass responses and the half-lives of the antibodies directed against these antigens, but this knowledge is important to evaluate the effect of using merozoite antigens as vaccines [19, 39–42].

## Methods

### Study area and participants

A longitudinal study was conducted in Igbo-Ora, a rural town in Nigeria. Malaria is endemic with higher transmission during the rainy season (April to October) with a mean seasonal entomological inoculation rate (EIR) of 131 [43]. Individuals (200) between five and 70 years old were enrolled in the study from July 2009 to July 2010. Venous blood samples were collected at baseline for blood group typing, blood genotyping and immunological studies. Thick and thin smears were made for parasitological investigations. The participants were followed up clinically and parasitologically at least once a month for a period of eight months. Only participants who were permanent residents were included in the study. Pregnant women, children under the age of five, and those with signs of severe hepatic or renal dysfunction, sickle cell disease, G6PD or seropositivity for HIV were excluded. Participants who fell ill with malaria were treated with oral artemisinin-lumefantrine for clinical malaria and blood films were monitored until negative. Children diagnosed for severe malaria were admitted to hospital and treated with i/v quinine according to WHO recommendations. Written informed consent by adult participants or parent/guardian was received, and ethical permissions were granted for the study from University of Ibadan and Ethics Committee in Nigeria (UI/IRC/06/0038) and the Stockholm Ethical Review Board (2013/4:8). From the original 200 individuals that were recruited for the longitutinal study, 40 were randomly selected to be used in this study, with a total of 302 samples used. Eighteen of the individuals were children (aged five to 13 years, 17 were parasite positive at some stage during the eight-month sample collection time) and 22 were adults (21 were parasite positive).

### Recombinant proteins

The recombinant merozoite antigens used in this study have been described in detail previously [44]. In brief, regions III to V of EBA175 (3D7; aa 761 to 1298) [19] and PfRh2A9 (3D7; aa 2027 to 2533, common region for both PfRh2a and PfRh2b) [42] were expressed in *E. coli* as GST-tagged fusion proteins.

### Measuring total IgG and subclasses against recombinant antigens

Enyme-linked immunosorbent assay (ELISA) for total IgG against EBA175 and PfRh2A9 were performed as described previously [41]. The antibody reactivity against subclasses IgG1-IgG4 was measured as follows: Flat bottom, 96-well plates (Nunc-immunoplate, Thermo Scientific) were coated with 1 μg/ml recombinant antigen in coating buffer (15 mM Na_2_CO_3_ and 35 mM NaHCO_3_; pH 9.6), washed 3x with PBS Tween 20 (PBS with 0.05% v/v Tween 20), blocked with 10% skimmed milk (Sigma-Aldrich M7409) in PBS-Tween 20 and washed 3x. Plasma samples diluted in 5% skimmed milk PBS-Tween 20 (1:25) were incubated for 1.5 hours (37°C), washed 3x, and mouse anti human antibodies were added (IgG1 A10630, IgG2 05-3500, IgG3 05-3600, IgG4 A10651 (all from Invitrogen Corp, CA, USA)) and diluted in 5% skimmed milk in PBS-Tween 20 (1/500 for IgG1 and IgG3, and 1/250 for IgG2 and IgG4, dilutions chosen after optimization) and incubated for 1.5 hours at 37°C. After washing (3x) goat anti-mouse IgG (H + L) horseradish peroxidase (HRP) (G21040, Invitrogen Corp, CA, USA) was added at 1/500 for IgG1 and IgG3, and at 1/1,000 for IgG2 and IgG4, incubated for 1.5 hours at 37°C, washed 3x and antibody reactivity was detected with azino-bis(3-ethylbenthiazoline-6-sulfonic acid) (ABTS) tablet (Sigma Aldrich) dissolved in phosphate citrate buffer pH 5.0 (Sigma Aldrich); 30% H_2_0_2_ was added just before use. Plates were incubated at room temperature for 1 hour and optical density (OD) read at 414 nm. All assays were run in duplicate and OD was subtracted for non-specific binding of the fusion tag, GST. Positive (pools of immune samples) and negative (Swedish non-immune) controls were included on all plates to allow for standardization. Non-immune samples gave OD values below 0.06. Antibody reactivity was considered positive when the absorbance was greater than the mean plus three standard deviations of the value for the non-immune Swedish samples.

### Statistical analyses

Data analyses were performed using GraphPad Prism Version 5.0a software. Antibody levels were compared by using Mann-Whitney t-test or ANOVA. The Spearman rank correlation was used to assess the association between two continuous variables. Two-tailed P-values were considered significant if they were <0.05. Data analyses were performed on data from time point follow-up during the study period. Antibody levels and subsequent levels of parasitaemia over the whole study period were analysed.

Under the assumption that antibodies decay at a constant rate, the half-lives of antibody decay were estimated for individuals where decreasing antibody levels were observed for at least three consecutive samples. The rate of antibody decay and half-life was estimated via linear regression of log antibody titre against the time of sampling.

## Results

### Total IgG and IgG subclass responses to EBA175 and PfRh2

**Figure 1:**
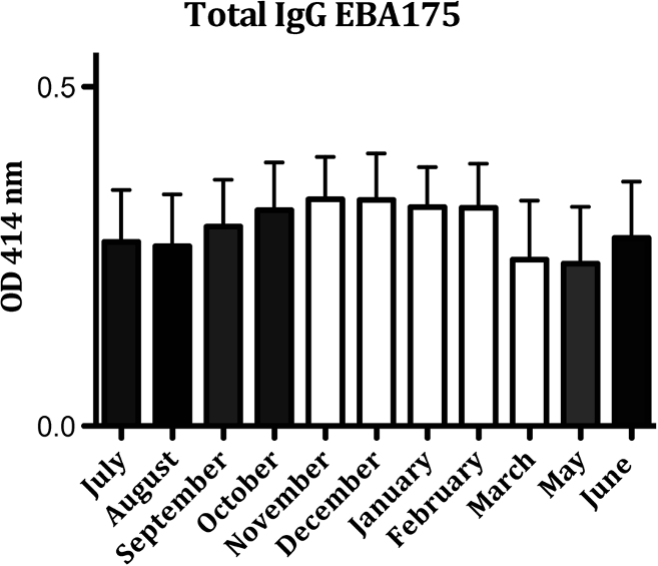
**Total IgG responses to EBA175 in individuals five to 70 years old (number of individuals = 40) from July 2009 to June 2010 (number of samples = 302).** The bars show mean ± SEM optical density (OD) value at 414 nm. (April not shown, no sample collection). (Black bars = rainy season; white bars = dry season). PfRh2: Data not shown.

IgG and subclass responses to the recombinant merozoite antigens EBA175 and PfRh2, were measured by ELISA. For EBA175, the total IgG levels in the study group increased from August with peak in November (followed by decline in the following months (Figure 1). The IgG levels for PfRh2 showed a similar pattern, and when the different subclass responses (IgG1-4) for both EBA175 and PfRh2 were studied, the fluctuations over time were similar to the values for total IgG. All patterns were also similar whether children or adults were investigated separately or together. All 40 individuals included were tested between seven and eight times during the study period. All individuals (100%) had IgG antibodies against EBA175 and PfRh2 at some stage during the eight months of follow-up (Table 1).

**Table 1:** **The prevalence of antibodies against EBA175 and PfRh2 during the study period**

Prevalence (%) ^a^		PfRh2		EBA175
	5-13 years	14-70 years	5-13 years	14-70 years
	(18 children)	(22 adults)	(18 children)	(22 adults)
**Total IgG**	100%	100%	100%	100%
**IgG1**	83%	95%	94%	82%
**IgG2**	86%	86%	94%	82%
**IgG3**	83%	77%	94%	91%
**IgG4**	89%	77%	94%	95%

^a^= Positive prevalence when antibody titers (OD) were greater than the mean plus 3 SD of the value for the non-immune Swedish donors.

**Figure 2:**
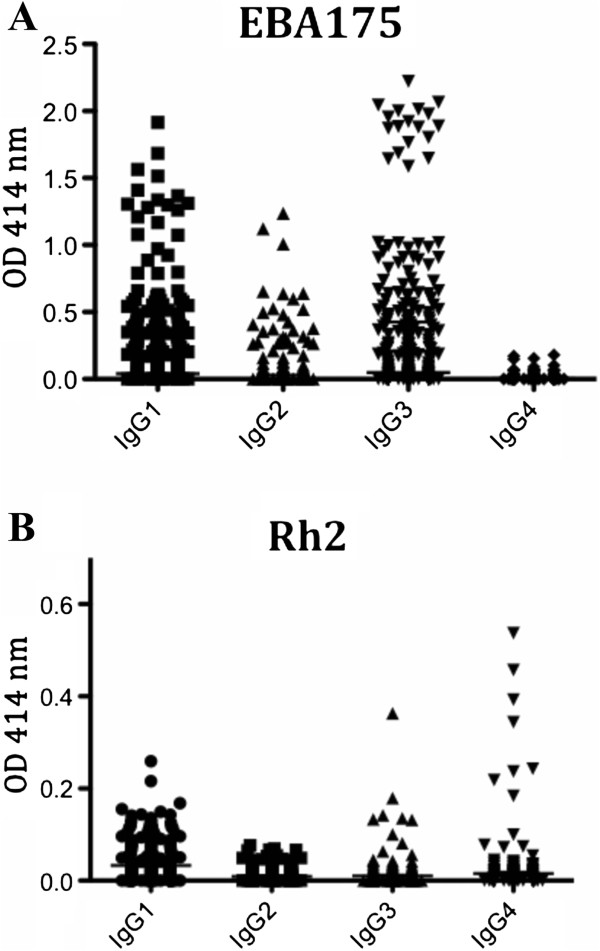
**IgG subclass responses to** ***Plasmodium falciparum*** **merozoite antigens (A) EBA175 and (B) PfRh2 in individuals five to 70 years old, followed over almost one year.** The scatter plot shows mean ± SEM OD for values at 414 nm; all samples (n = 302) from all individuals (n = 40) are included.

When the subclass responses were studied, it was noted that for both EBA175 and PfRh2 the response was dominated by the cytophilic IgG1 and IgG3, but there was also some IgG2 response (Figure 2). For PfRh2 there was also a relatively strong response for IgG4. When the values were correlated to each other, it was found that total IgG levels against EBA175 and PfRh2 showed a weak to moderate, but significant positive correlation with each other (*R*^2^ = 0.22, *p* = 0.0002), and this was also noted for the IgG1 levels (*R*^2^ = 0.25 and *p* <0.0001), indicating co-acquisition of different antibodies with malaria exposure. No significant correlations were observed in IgG2, IgG3 or IgG4 between EBA175 and PfRh2 OD values (IgG2, *R*^2^ = 0.1 and *p* = 0.1; IgG3, *R*^2^ = 0.05 and *p* = 0.4; IgG4, *R*^2^ = 0.02 and *p* = 0.7). Furthermore, when the total IgG and subclass responses within EBA175 and PfRh2 were investigated it was observed that there were good correlations for EBA175, but less so for PfRh2 (Table 2).

**Table 2:** **Correlations of total IgG and subclass IgG1-IgG4 responses against EBA175 and PfRh2**

Antigen-IgG	Spearman r	***p***-value
**EBA175 Total IgG vs**		
**IgG1**	0.57	**<0.0001**
**IgG2**	0.46	**<0.0001**
**IgG3**	0.49	**<0.0001**
**IgG4**	0.38	**<0.0001**
**EBA175 IgG1 vs**		
**IgG2**	0.5	**<0.0001**
**IgG3**	0.63	**<0.0001**
**IgG4**	0.25	**<0.0001**
**EBA 175 IgG2 vs**		
**IgG3**	0.48	**<0.0001**
**IgG4**	0.40	**<0.0001**
**EBA175 IgG3 vs**		
**IgG4**	0.34	**<0.0001**
**Rh2 Total IgG vs**		
**IgG1**	0.55	**<0.0001**
**IgG2**	0.07	0.26
**IgG3**	0.04	0.48
**IgG4**	0.1	0.08
**Rh2 IgG1 vs**		
**IgG2**	0.07	0.23
**IgG3**	0.09	0.13
**IgG4**	0.1	0.07
**Rh2 IgG2 vs**		
**IgG3**	0.27	**<0.0001**
**IgG4**	0.21	**0.004**
**Rh2 IgG3 vs**		
**IgG4**	-0.05	0.35

When male and female subjects were analysed separately, samples from females showed slightly higher values for total IgG (p = 0.0025), IgG3 (p = 0.04) and IgG4 (p = 0.03) for EBA175, and IgG2 (p = 0.02) and IgG4 (p = 0.006) for PfRh2. Male individuals showed higher values only for IgG1 EBA175 (p = 0.03).

### Correlation of antibody responses with age

When age was correlated to the levels of antibodies, it was found that there were significant positive correlations with total IgG (*R*^2^ = 0.12 and *p* = 0.036), IgG1 (*R*^2^ = 0.29 and *p* = <0.0001) and IgG3 (*R*^2^ = 0.33 and *p* <0.0001) for EBA175. IgG2 and IgG4 against EBA175 did not show any correlations with age (IgG2, *R*^2^ = 0.01, p =0.8; IgG4*, R*^2^ = 0.03, p = 0.6)*.* There were no significant correlations between age and levels of antibodies against PfRh2 (*p*-values 0.3-0.8*).*

### Correlation of antibody responses with parasitaemia

**Figure 3:**
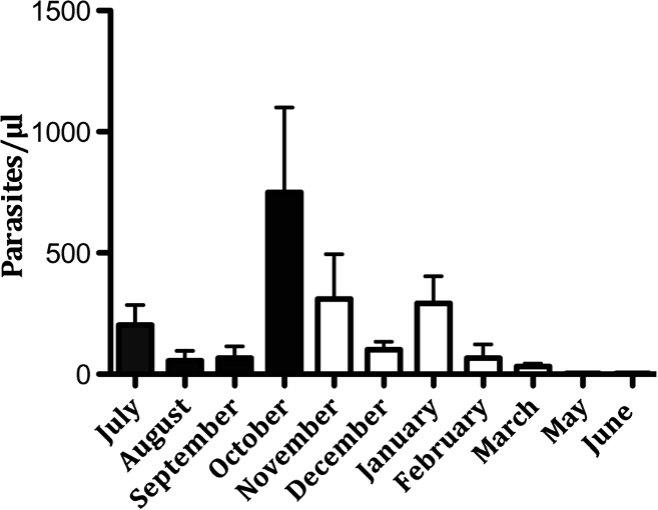
**Parasitaemia in individuals (n = 39) five to 70 years old from July 2009 to June 2010.** The bars show mean ± SEM parasitaemia (parasites/μl). (April not shown, no sample collection; June: one individual who had very high parasitaemia, 15 168 parasites/μl, was excluded from this figure. (Black bars = rainy season, white bars = dry season).

The parasitaemia in the study group was monitored during the whole study period. The highest peak in parasitaemia was observed in the month of October (mean: 749.7 parasites/μl), while the lowest number was seen in May (mean: 3.7 parasites/μl) (Figure 3). There was no significant difference in parasitaemia between children (5-13 years) and adults (14-70 years) (p = 0.3).

To assess whether the antibody responses in this study group were associated with protection, the relationship between antibody levels and parasitaemia was investigated. Higher IgG1, IgG2 and IgG3 levels for EBA175 and total IgG for PfRh2 correlated significantly with lower parasitaemia (*R*^2^ = 0.19, *p* = 0.002; *R*^2^ = 0.19, *p* = 0.002; *R*^2^ = 0.21, *p* = 0.0008; *R*^2^ = 0.24, *p* <0.0001, respectively). There were no significant differences between IgG levels measured before and after an episode of parasitemia (not shown).

Additionally, individuals with the highest quartiles of levels of total IgG against EBA175 or PfRh2 showed an even higher correlation with reduction of parasitaemia (EBA175, *R*^2^ = 0.54, *p* <0.0001; PfRh2, *R*^2^ = 0.42, *p* = 0.002, Spearman rank correlation comparing levels of antibodies with parasitaemia for every sample), and when individuals with high total levels of both antibodies against EBA175 and PfRh2 were considered (number of individuals = 11, number of samples = 78) they had a lower parasitaemia compared to those with raised levels of only one of the antibodies (*p* = 0.03).

### The impact of HbAA and HbAS carriage on the levels of IgG

Out of the 40 patients included in this study, 30 had HbAA (normal, adult haemoglobin), nine had HbAS and one had HbAC. There was no significant difference in parasitaemia or age between the HbAA and HbAS groups (p = 0.27 and 0.32, respectively).

When all individuals were considered together, total IgG, IgG1 and IgG3 against EBA175 were significantly higher in individuals with HbAA compared to HbAS (IgG, *p* = 0.008; IgG1, *p* = 0.048; IgG3, *p* = 0.0001). For PfRh2, only the IgG1 response was significantly higher in HbAA (*p* = 0.0001).

**Figure 4:**
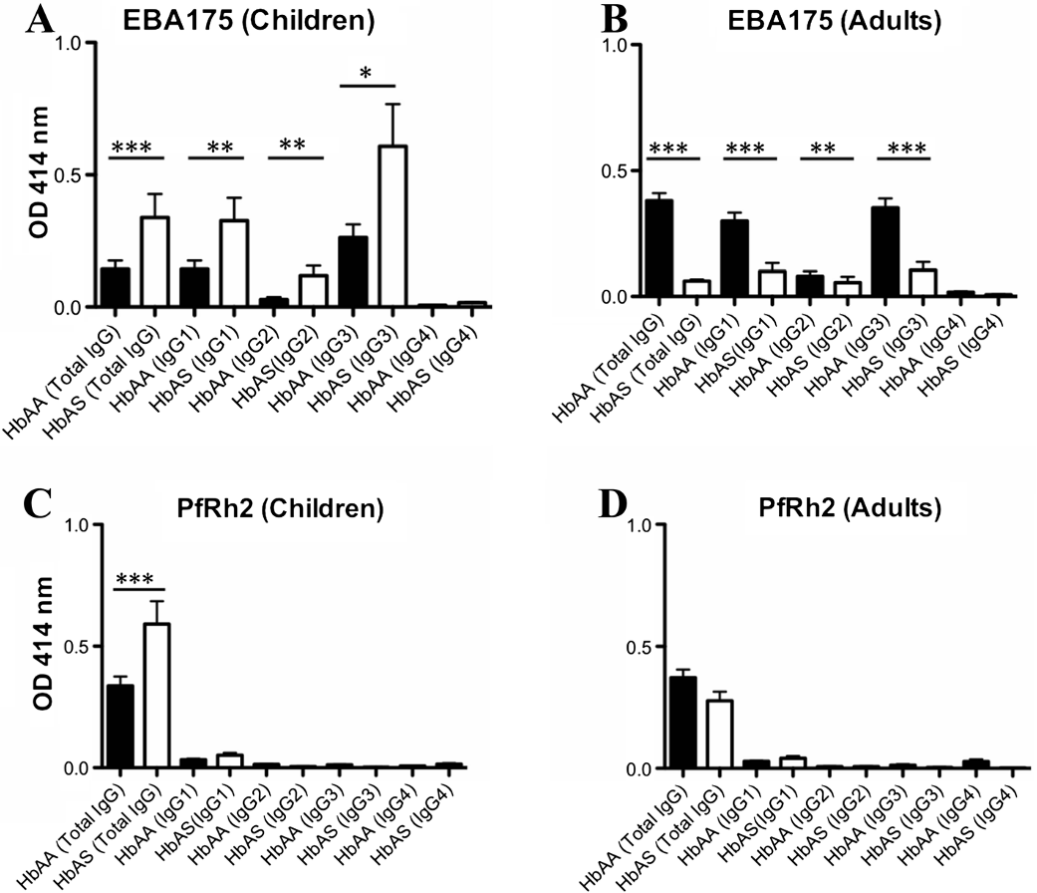
**The total IgG and subclass levels to EBA175 (A, B) and PfRh2 (C, D) in HbAA and HbAS for children (n = 18) and adults (n = 22) are shown; the number of samples = 302.** The bars show mean ± SEM of OD at 414 nm. (*, *p* <0.05; **, *p* <0.01; ***, *p* <0.001).

When the samples where divided into the two groups children (n = 18, age 5-13) and adults (n = 21, age 14-70), children with HbAS had significantly higher levels of IgG, IgG1, IgG2, and IgG3 against EBA175 and higher levels of IgG against PfRh2 compared to HbAA (Figure 4). When adults were considered, the pattern was the opposite with higher levels of IgG, IgG1 and IgG3 against EBA175 in HbAA individuals (there were no significant differences for PfRh2).

### Comparison of half-lives of total IgG and subclasses

**Figure 5:**
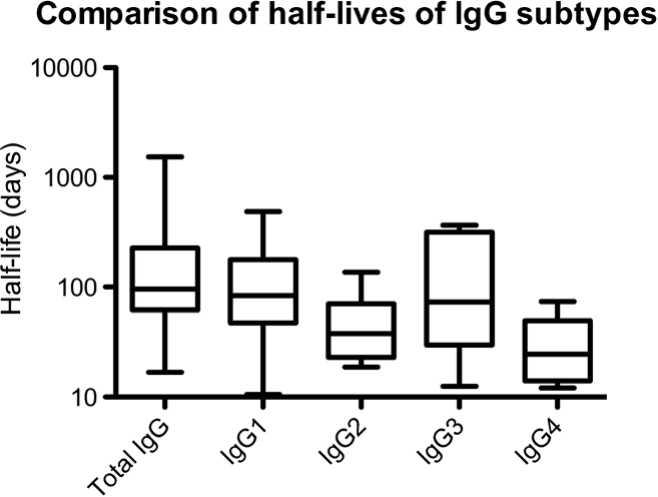
**The half-lives of IgG2 and IgG4 were shorter than those for IgG1 and IgG3.**

For EBA175, IgG2 and IgG4 showed significantly shorter half-lives compared to IgG1 (p <0.05/<0.01), to IgG3 (p <0.05/<0.01), and to total IgG (p <0.01 for both) (Figure 5). For PfRh2, a similar trend was seen as for EBA175, but the only significant differences seen were those that showed shorter half-lives for IgG1, IgG2 and IgG4 compared to total IgG, and a shorter half-life for IgG4 compared to IgG1 (p <0.05 for all comparisons). There were no significant associations between half-lives and ages of the tested individuals.

For total IgG, half-lives of parasite positive and parasite negative individuals were compared. For EBA175, the period of antibody decay was 98 days for parasite positive individuals and 64 days for parasite negative individuals. For PfRh2, the half-life was 120 days for parasite positive and 82 days for parasite negative individuals. However, due to the small number of parasite negative individuals, it is difficult to estimate the value of these differences.

## Discussion

In this study, total IgG and subclass responses against EBA175 and PfRh2 were investigated, and the effects of HbAS in children and adults living in Igbo-Ora, a rural area of Nigeria, over a period of almost one year. These antigens were chosen because they are potential vaccine candidates, and they use different invasion pathways. EBA175 uses a sialic acid (SA)-dependent invasion pathway with glycophorin A as its main receptor. PfRh2 plays a role in SA-independent invasion (with an as yet unknown receptor ‘Z’) [4], but some data suggest PfRh2 can also bind SA on the erythrocyte surface [45, 46]. The EBAs and PfRhs play a vital role in merozoite invasion [47]. It has been suggested that a vaccine could be more efficient if antigens from different invasion pathways are used [38, 48], and it is therefore of interest to study the combined response in individuals who have acquired immunity by natural exposure to the *P. falciparum* parasite. Very few studies have investigated the subclass response to merozoite antigens over time.

The response for each subclass approximately followed the pattern of total IgG for both EBA175 and PfRh2, with a peak in antibody levels arising just after the peak in parasitaemia. This goes in line with the transmission intensity being highest during the rainy season, which ends in October. IgG1, IgG2 and IgG3 for EBA175 were positively associated with lower parasitaemias, indicating that all of these antibodies are possibly important for protection against malaria. These findings are in line with earlier studies that showed high levels of IgG, including IgG1 and IgG3 to EBA175, to be associated with protection from malaria [19, 49, 50]. Individuals with high levels of both total IgG against EBA175 and PfRh2 had a lower parasitaemia compared to those that had high levels of only one of the antibodies. This observation of combined antibody responses against two antigens underlines the importance of using combinations of antigens in vaccine trials [38, 48]. When results from male and female individual were analysed, they were often slightly higher levels of antibodies in women. The reason for this is not clear, perhaps it is because of slightly different exposure.

In the studies that have been done before, looking at subclass responses against merozoite antigens, the responses have been dominated by IgG1 and IgG3 [19–21, 25, 42, 51–53]. However, in this study, there was some response against both IgG2 and IgG4. IgG1 and IgG3 are considered cytophilic [23, 51] but in malaria non-cytophilic antibodies could also be of major importance. Even though the levels are low, the quality of the antibodies might be of more importance [54]. A study showed that high levels of IgG2 against RESA and MSP2 were associated with a lower risk of infection, indicating that IgG2 may be important, while IgG4 was suggested to block the cytophilic activity [22]. IgG4 has also before been shown to be of doubtful effect, in the response against MSP1-19, and it is especially interesting since this study was done in the same area of Nigeria as this study [55]. Omosun *et al*. [55] found dominating IgG1 and IgG3 responses to MSP1-19. While both IgG2 and IgG4 against MSP1-19 correlated positively with age, there was also a positive correlation of parasite density with IgG2 and IgG4 levels. Even though relatively high levels of IgG4 (especially against PfRh2) was found, there was no protective effect of this subclass when associated with parasitaemia. In an earlier study of subclass responses in children against PfRh2, mostly IgG1 and IgG3 were seen [42]. The reason for why the results from this study are slightly different might be because the population is different (Papua New Guinea compared to Nigeria), but also because all ages were included in this study. To elucidate whether IgG4 is protective or actually counter-acting protection, further studies are needed, but this study indicates that more subclass analysis should be included in studies of immunity in malaria.

For EBA175, IgG2 and IgG4 showed significantly shorter half-lives compared to IgG1 and IgG3. This is interesting since the half-life of IgG3 is normally considered to be considerably shorter than the other subclasses [56]. The short half-life noted here for IgG2 and IgG4 might help in explaining why IgG1 and IgG3 are often considered the most important ones in protection against malaria. It was also noted that parasite positive individuals had longer half-lifes of their IgG for both EBA175 and PfRh2, compared to individuals who were parasite negative all throughout the study period, which makes sense since the parasite positive individuals are exposed to parasites under a longer time period.

In this Nigerian population, the response in general towards EBA175 seemed stronger than against PfRh2. The subclass IgG1-IgG4 response against PfRh2 was low in this group. It has been shown before [45] that the expression of PfRh2 can vary between parasites, and it is not known which parasites were circulating in the area at the time. It might be that the recombinant antigen of EBA175 was more similar to the circulating parasites than the version of PfRh2 that was used. The results might also vary depending on which part of the protein was used. Region III-V of EBA175 was used in this study [19], and previous studies suggest that antibodies to region II of this protein have a lower protective association [13]. Antibodies to EBA and PfRh invasion ligands are thought to act primarily by inhibiting erythrocyte invasion, and studies have shown that vaccine-induced antibodies in animal studies [57, 58], and in humans for EBA175 [41, 59], can inhibit parasite growth *in vitro*. In previous studies of Kenyan children and adults, there has been evidence for that EBA175 and other EBAs, and PfRh invasion ligands are important targets of inhibitory antibodies, and variation in the function of these ligands can facilitate evasion of inhibitory antiobodies [41, 54]. Recent studies have also demonstrated that affinity-purified human antibodies to EBA175, PfRh2, PfRh4, and PfRh5 can effectively inhibit invasion *in vitro* [59–62]. As a continuation of the studies in this paper, there are plans to compare invasion inhibition results to the subclass responses to elucidate whether any of the IgG subclasses could be more inhibitory than others.

When individuals with different haemoglobin were compared, there were different responses in children and adults, with higher levels of antibodies for HbAS in children. Children with HbAS had significantly higher levels of IgG, IgG1-3 against EBA175, and higher IgG levels against PfRh2. For adults the pattern was the opposite with higher IgG and IgG1-3 levels against EBA175, and higher IgG against PfRh2 in HbAA. It has been suggested before that the haemoglobin can affect the antibody response in malaria, but then the discussion has mainly concerned surface molecules on the erythrocyte [63, 64]. Previous studies in Nigeria [65] and in other African countries [30, 66, 67] have shown lower parasite densities as well as lower prevalence of both uncomplicated and severe malaria in individuals with HbAS compared to HbAA. Even though this study is based on a relatively small number of samples, the results indicate that further studies on differences between HbAS and HbAA individuals should be done for merozoite antigens and not only erythrocyte surface antigens.

## Conclusions

Subclasses of IgG against both EBA175 and PfRh2 could be protective against malaria since they correlated with a lower parasitaemia. There were shorter half-lives of IgG2 and IgG4 against EBA175, which might explain why these subclasses are usually considered to be less important in protection against malaria. In evaluation of vaccine studies it might be important also to consider haemoglobin polymorphisms in different populations, especially in an area where HbAS is relatively common.
